# A Review of the Diagnosis and Treatment of Limbal Stem Cell Deficiency

**DOI:** 10.3389/fmed.2022.836009

**Published:** 2022-05-25

**Authors:** Anahita Kate, Sayan Basu

**Affiliations:** ^1^The Cornea Institute, KVC Campus, LV Prasad Eye Institute, Vijayawada, India; ^2^The Cornea Institute, KAR Campus, LV Prasad Eye Institute, Hyderabad, India; ^3^Prof. Brien Holden Eye Research Centre (BHERC), LV Prasad Eye Institute, Hyderabad, Telangana, India

**Keywords:** Limbal stem cell deficiency (LSCD), simple limbal epithelial transplantation (SLET), limbal stem cell transplantation (LSCT), Keratoprosthesis (KPro), Anterior segment optical coherence tomography (AS-OCT), impression cytology (IC), confocal microscopy, cultivated limbal epithelial transplantation (CLET)

## Abstract

Limbal stem cell deficiency (LSCD) can cause significant corneal vascularization and scarring and often results in serious visual morbidity. An early and accurate diagnosis can help prevent the same with a timely and appropriate intervention. This review aims to provide an understanding of the different diagnostic tools and presents an algorithmic approach to the management based on a comprehensive clinical examination. Although the diagnosis of LSCD usually relies on the clinical findings, they can be subjective and non-specific. In such cases, using an investigative modality offers an objective method of confirming the diagnosis. Several diagnostic tools have been described in literature, each having its own advantages and limitations. Impression cytology and *in vivo* confocal microscopy (IVCM) aid in the diagnosis of LSCD by detecting the presence of goblet cells. With immunohistochemistry, impression cytology can help in confirming the corneal or conjunctival source of epithelium. Both IVCM and anterior segment optical coherence tomography can help supplement the diagnosis of LSCD by characterizing the corneal and limbal epithelial changes. Once the diagnosis is established, one of various surgical techniques can be adopted for the treatment of LSCD. These surgeries aim to provide a new source of corneal epithelial stem cells and help in restoring the stability of the ocular surface. The choice of procedure depends on several factors including the involvement of the ocular adnexa, presence of systemic co-morbidities, status of the fellow eye and the comfort level of the surgeon. In LSCD with wet ocular surfaces, autologous and allogeneic limbal stem cell transplantation is preferred in unilateral and bilateral cases, respectively. Another approach in bilateral LSCD with wet ocular surfaces is the use of an autologous stem cell source of a different epithelial lineage, like oral or nasal mucosa. In eyes with bilateral LSCD with significant adnexal issues, a keratoprosthesis is the only viable option. This review provides an overview on the diagnosis and treatment of LSCD, which will help the clinician choose the best option amongst all the therapeutic modalities currently available and gives a clinical perspective on customizing the treatment for each individual case.

## Introduction

The corneal epithelium is essential for the maintenance of the anatomic integrity and physiological functioning of the transparent cornea. The maintenance of the corneal surface is ensured by the constant turnover of the corneal epithelium from the limbal epithelial stem cells (LESC) (1, 2). These LESC straddle the junction between the cornea and the conjunctiva and reside in the basal epithelial layer of the limbus. The microenvironment surrounding the LESC within the palisades of Vogt, is responsible for ensuring the viability and efficacy of the stem cells. The LESC prevent the migration of the conjunctival epithelial cells over the corneal surface and in the presence of a dysfunction of the LESC themselves or the surrounding niche, there occurs conjunctivalization of the cornea.

Limbal stem cell deficiency (LSCD) can stem from numerous etiologies, resulting in serious visual morbidity (3, 4). And so, early diagnosis of this entity is essential in order to institute the appropriate therapy in a timely manner. Also, the need for diagnosing LSCD is even more essential when a keratoplasty is planned as the graft is unlikely to fair well if the LSCD is not corrected in advance. Although the diagnosis of LSCD is still primarily a clinical one, there are several diseases that can mimic its clinical picture (5, 6). In such scenarios, the clinician can choose from an array of diagnostic tests aimed at detecting LSCD. Similarly, numerous therapeutic options are available in management of LSCD and the choice of one intervention over the other depends upon the severity of ocular and adnexal involvement. This review aims to provide an understanding of the various tools in the diagnostic armamentarium of LSCD in the context of their advantages and limitations. It also endeavors to crystallize the clinical approach to a case of LSCD based on the laterality, severity, and resources available.

## Etiology

Pathologies that affect the LESC or their supporting niche can cause LSCD (3). These can be classified as per Table 1. Understanding the underlying primary disease process often provides an added perspective into the management of LSCD. Several conditions such as chemical or thermal ocular burns, Stevens-Johnson syndrome (SJS), etc. are one-time insults and usually the treatment approaches are limited to the sequalae that ensue (7). On the other hand, in autoimmune disorders such as mucous membrane pemphigoid (MMP), there is a constant disruption of the systemic and ocular milieu occurring via inflammatory mediators (8). In such cases, addressing the LSCD in isolation invariably has very poor outcomes and so it must be done in conjunction with the management of the systemic pathology. Furthermore, in case of congenital causes of LSCD, treatment options include specific gene targeted therapy which is possible only if a particular type of limbal stem cell transplant (LSCT) is performed. Therefore, it is essential for the treating physician to know the primary disease process in order to make an informed decision and choose the appropriate therapeutic modality on a case-to-case basis.

**Table 1:** Causes of limbal stem cell deficiency.

Congenital
Congenital aniridia
Multiple endocrine deficiency
Ectodermal dysplasia
Epidermolysis bullosa
Xeroderma pigmentosum
Traumatic/Acquired
Ocular burns (Chemical/thermal)
Post-surgical
Contact lens wear
Radiation
Drug Induced
Autoimmune
Stevens-Johnson syndrome
Mucous membrane pemphigoid
Sjogren's syndrome (Primary and Secondary)
Vernal keratoconjunctivitis
Graft-vs. host disease
Idiopathic

## Clinical Features

### Symptoms

Patients with LSCD present with non-specific symptoms such as ocular redness, discomfort, pain, watering, and photophobia. When the disease is severe enough to involve the visual axis, the complaints extend to blurring or decreased vision (2, 7).

### Signs

**Figure 1:**
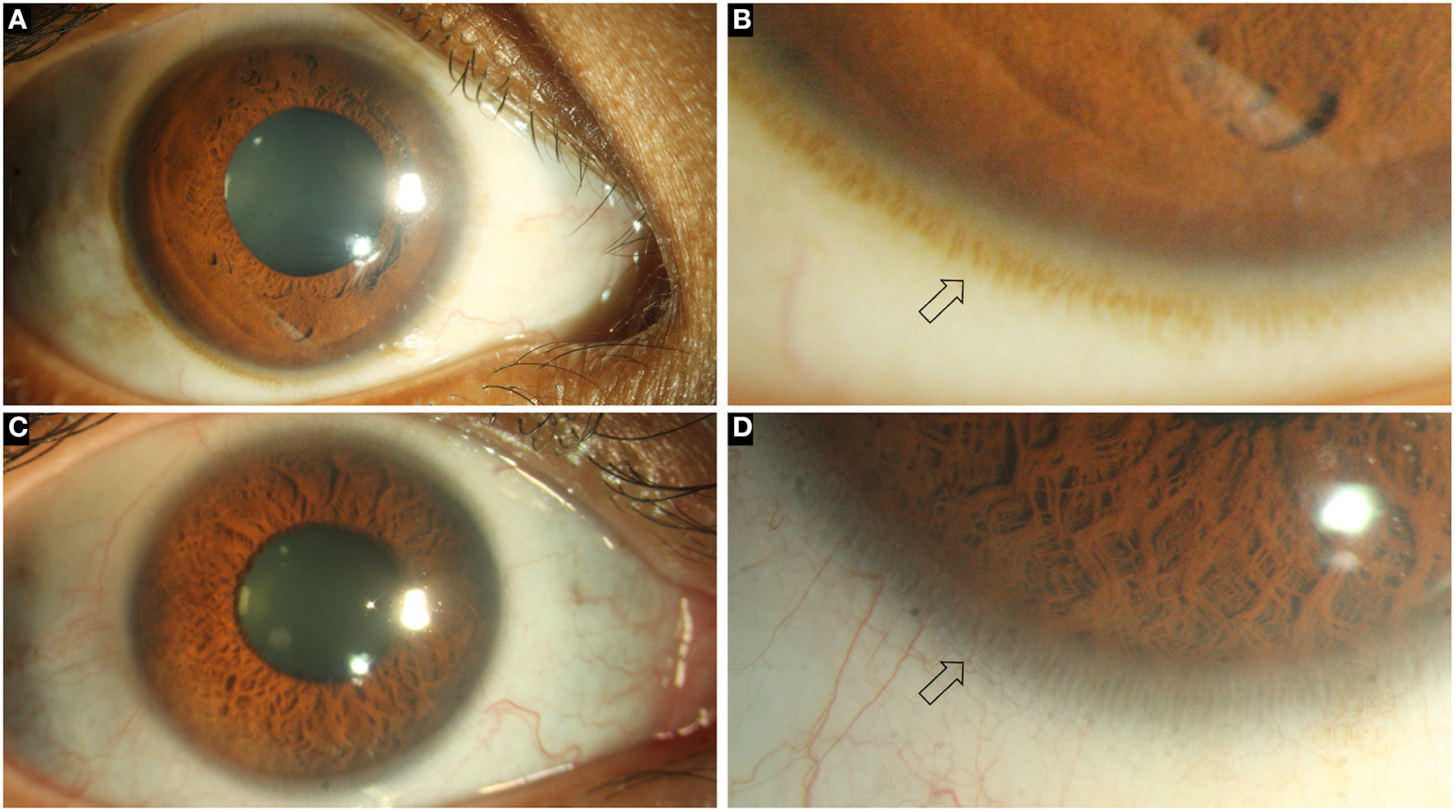
Collage of images depicting the normal ocular surface and limbus (arrows) in pigmented **(A, B)** and hypopigmented **(C, D)** eyes.

**Figure 2:**
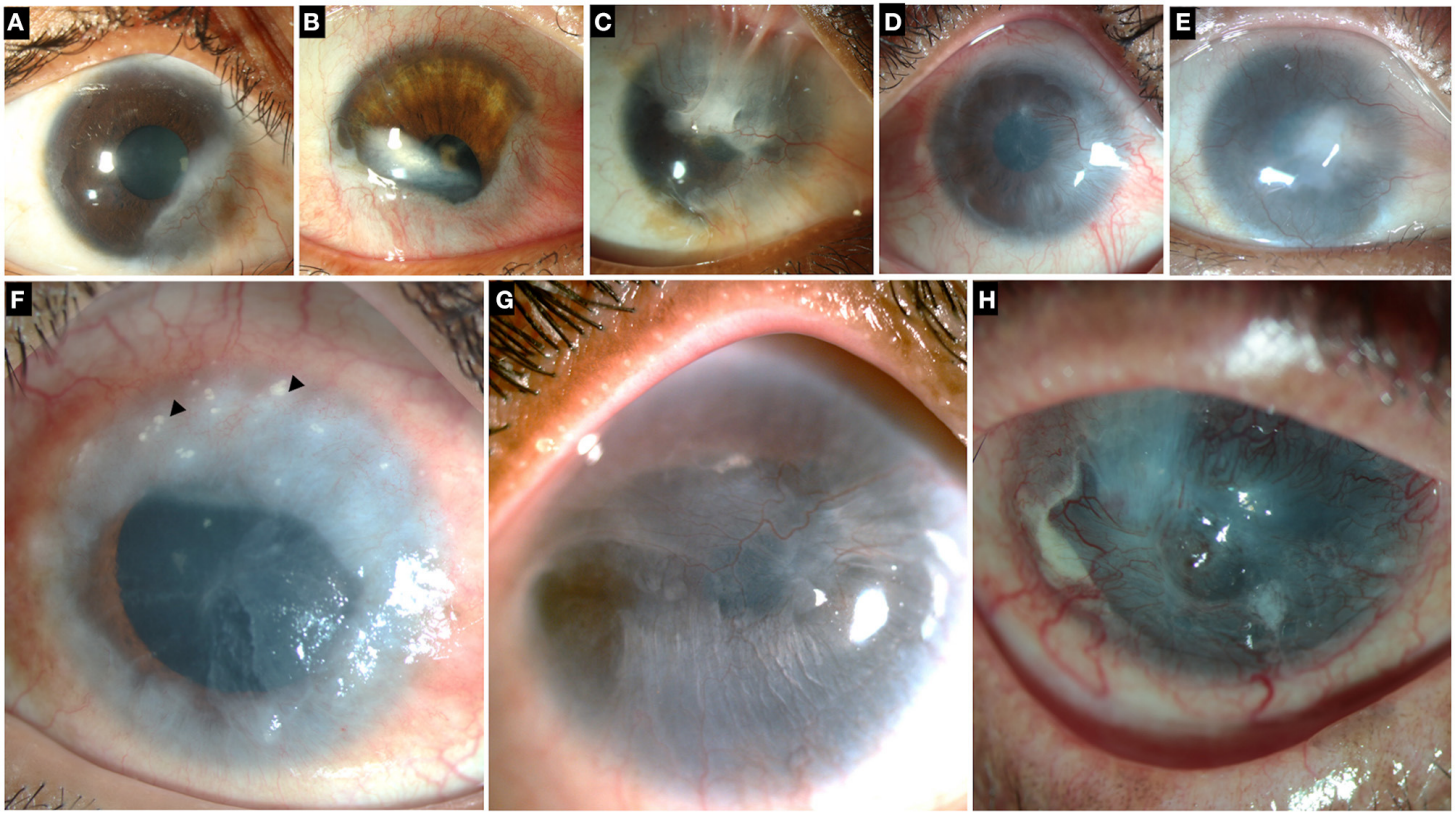
Collage of images illustrating different grades and etiologies of limbal stem cell deficiency (LSCD). Top row: LSCD due to chemical injury which is partial and sparing the visual axis **(A)**, involving the visual axis **(B,C)**. **(D,E)** Total LSCD in chemical injury. **(F)** LSCD in chronic vernal keratoconjunctivitis. Superior cornea shows Horner-Trantas dots (black arrowheads). **(G)** LSCD in Epidermolysis Bullosa **(H)** LSCD in mucous membrane pemphigoid.

**Figure 3:**
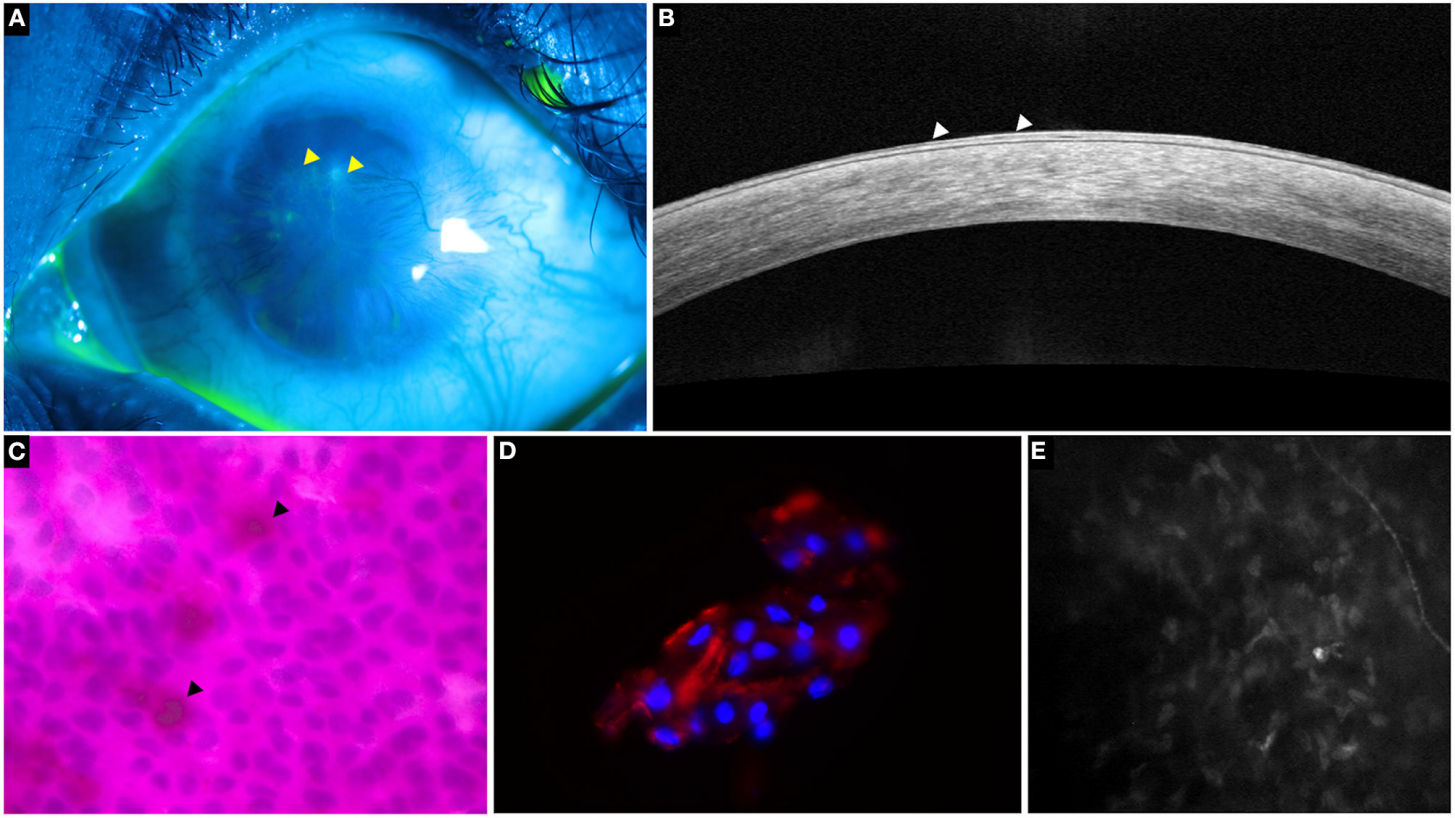
A representative collage of various diagnostic modalities in limbal stem cell deficiency (LSCD). **(A)** Fluorescein-stained image showing characteristic stippled staining (yellow arrowheads). **(B)** Optical coherence tomography line scan showing hyperreflective epithelium indicative of LSCD (white arrowheads). **(C)** Impression cytology depicting Periodic acid-Schiff positive goblet cells (black arrowheads) and CK19 positive cells on immunohistochemistry **(D,E)** *in vivo* confocal microscopy showing decreased sub-basal nerve density.

The diagnosis of LSCD is primarily clinical but needs to be confirmed by one or more objective methods. The clinical findings vary depending upon the severity of the disease. In early cases of LSCD, there may be focal areas of the corneal epithelium which take up the characteristic stippled staining pattern (7). There is loss of clarity within the epithelium, creating a lackluster appearance. The limbal palisades of Vogt, which are usually most easily visible superiorly and inferiorly, may be difficult to discern or may become flattened (Figure 1). With the progression of the disease there occurs conjunctivalization of the cornea and superficial corneal vascularization (Figure 2) (7, 8). Due to patches of irregular epithelial thinning, a whorl pattern is noted which is better picked up as areas of pooling up of fluorescein(Figure 3). These zones also exhibit late staining (7, 8). A sharp demarcation between the abnormal and normal corneal epithelium may also be seen in cases of sectoral involvement (7–9). Epithelial instability is a hallmark of the disease process which manifests as repeated breakdown of the epithelium and in advanced cases this can progress to form a persistent epithelial defect (PED) (7). Recurrent episodes of PEDs can affect the underlying stroma leading to scarring or sterile melts in non-resolving cases (7).

## Diagnostic Investigations

In cases of severe ocular burns or advanced cicatricial conjunctivitis following SJS, the diagnosis of LSCD can be straightforward. However, in several cases the clinical presentation is subtle and establishing the diagnosis may be challenging. In such cases the ancillary tests mentioned below help supplementing the diagnosis. In addition to confirming the diagnosis, these tests may facilitate the quantification of the disease and provide an understanding of its progression. They also help to confirm the epithelial phenotype following a stem cell transplant and in monitoring the postoperative recovery (10–13).

### Impression Cytology

This test involves sampling of the superficial epithelial cells of the ocular surface and subjecting them to histopathological and immunohistochemistry tests. The sample can be obtained from the cornea or the conjunctiva and is usually acquired using a nitrocellulose or cellulose acetate filter paper (14). Although the test typically acquires the superficial corneal and conjunctival cells, repeated sampling in a particular area will facilitate access to the deeper layers as well (14). Following a standardized sampling technique is recommended as this will affect the quantity and quality of tissue obtained (7, 9, 14). Ensuring that the ocular surface is not too wet and that the pore size of the paper is adequate to collect the epithelial cells will also help in improving the yield (9, 15).

#### Histopathology

The cytology specimen procured undergoes histopathological processing with various stains such as hematoxylin and eosin (H&E), Giemsa, Periodic acid-Schiff, etc (14). These stains detect the presence of goblets cells which indicates the invasion of conjunctival epithelial cells over the surface of the cornea (14). Although the detection of goblet cells is considered the sine qua non of LSCD (Figure 3), its absence does not imply a healthy limbus. Also, there may be a decrease in the concentration of goblet cells due to the underlying disease process itself as is the case in SJS (16, 17). As mentioned earlier the sensitivity of the test is largely dependent on the sampling procedure. And so, assessment of the epithelial cells which are also concurrently sampled can enhance the detection rate of LSCD. However, the differentiation of corneal from conjunctival epithelial cells is not possible with the routine stains used and requires immunohistochemistry.

#### Immunohistochemistry

Several markers have been investigated and of these cytokeratin 12 has been found to be specific for the mature corneal epithelium (7, 18). Although cytokeratin 3 was also considered to be cornea specific, recent studies have found this marker in the conjunctiva also (19, 20). Cytokeratin 7, 13 and 19 are markers which are specifically expressed in conjunctival epithelial cells while mucin 5AC(MUC5AC) is used for the detection of goblet cells (Figure 3) (18, 20–22). However, negative MUC5AC staining has been noted despite positive conjunctival marker staining, signifying the low sensitivity of this marker (18). This fallacy has been subverted with the use of reverse transcriptase polymerase chain reaction test for the detection of MUC5AC which increases the test sensitivity to 98% (23).

Obtaining normal corneal cells through impression cytology is challenging because of the inherent adherence of the cells to each other and the underlying basement membrane. This is in contrast to the conjunctival cells which freely desquamate and so, the presence of an abundance of cellularity can itself indicate the presence of conjunctival cells (18, 20) Since conjunctivalization of the cornea is considered a hallmark of LSCD, the confirmation of conjunctival epithelial cells from a corneal cytology specimen has been deemed sufficient to diagnosis LSCD (Figure 3) (20). The subsequent presence of the cytokeratin 12 marker is used to quantify the disease which is considered mild or partial if the corneal marker can still be detected (20). The degree of the fluorescence exhibited by these markers has also been used to quantify the severity of the disease (19, 24).

### *In-Vivo* Confocal Microscopy (IVCM)

IVCM is a non-invasive tool that provides an *in vivo* picture of the microstructures within the cornea. Of the various parameters measured by the device, presence of goblet cells, the basal epithelial measurements of the cornea and limbus along with the changes of the sub-basal nerve plexus are used in the diagnosis of LSCD (Figure 3).

#### Goblet Cells

The presence of goblet cells in a corneal IVCM scan is confirmatory of the diagnosis of LSCD. The detection rate of goblet cells with IVCM closely correlates with that of impression cytology (25). However, as mentioned previously, several factors may affect the detection of goblet cells in a case of LSCD and with an IVCM this is further confounded by the small area that is scanned. Also, the described morphology of a goblet cell is variable with descriptions of both a hypo and hyper-reflective cytoplasm (26–28). Thus, although the detection of goblet cells is feasible with an IVCM, the test has low sensitivity.

#### Corneal and Limbal Epithelial Changes

A decrease in basal cell density (BCD) with an increase in the size of the cells is noted in patients with LSCD (29–31). This decrease corresponds with the severity of the disease and in advanced cases, there is significant alteration in the morphology of the cells with an increased number of visible hyperreflective cell nuclei (31, 32). Deng et al. found that a BCD value of <7930 cells/mm^2^ for basal cell density diagnosed LSCD with a 95.5% sensitivity and 100% specificity (31). In cases of partial LSCD, the epithelium in the clinically normal areas maintains the normal pattern on IVCM although there is often an increase in the number of dendritic cells in the underlying stroma (25, 33, 34). A clear demarcation is noted at the junction between the corneal and conjunctival epithelial cells as the two have very distinct morphological features on IVCM (33). Corneal basal cells have a dark cytoplasm with well-defined borders and are much smaller than the conjunctival cells. Intraepithelial cystic lesions with surrounding goblet cells have also been described in cases of LSCD (33). Overall thinning of the epithelium is seen in LSCD (35). A similar pattern of change is noted in the limbal epithelium as well with a decreased BCD which correlates with disease severity (34–36). In cases of partial LSCD, the clinically unaffected areas also exhibit the same changes indicating a pre-clinical method of detection of LSCD (34, 36).

#### Corneal Nerves Changes

A progressive decrease in the density of the sub-basal plexus of nerves is noted with increasing severity of the disease until a complete nerve drop out occurs (Figure 3) (29, 34, 37). Additionally, several other changes have also been reported which include decreased branch length, increased angulation of branching, increased tortuosity, etc (31, 37). A cut off for sub-basal nerve density of 53 nerves/mm^2^ resulted in an 87% sensitivity and 91.7% specificity for the diagnosis of LSCD (31). Caro-Magdaleno et al. found that the sub basal nerve density had an inverse association with conjunctivalization and a value of <17,215 μm/mm^2^ diagnosed LSCD with a 95.5% sensitivity and specificity of 90.6% (38).

### Anterior Segment Optical Coherence Tomography (AS-OCT)

AS-OCT is a non-invasive imaging tool that has low operator dependence and yields repeatable results. It has been used to augment the diagnosis of LSCD with its corneal and limbal epithelial measurements. Additionally, with the help of image processing software, the reflectivity from these measurements have been quantified. The role of the angiography feature of OCT for detecting LSCD has also been investigated.

#### Epithelial Changes

Similar to the IVCM findings, a decrease in both the corneal and limbal epithelial thickness has been observed with AS-OCT in eyes with LSCD (Figure 3) (30, 39). Although epithelial thinning is not specific to LSCD and is seen in disease entities such as keratoconus, dry eye, etc.; the degree to which the thinning occurs is different. A 20–30% thinning has been reported in eyes with LSCD, while in other disorders the thinning is <10% (35, 39, 40). Liang et al. proposed a new parameter measured as a mean of the central epithelial thickness and thickness measured at two points, 1 mm on either side of the central thickness (39). Values <46.6 μm for this parameter were considered diagnostic for LSCD with a sensitivity and specificity of 61.7% and 100% respectively (39).

In addition to measuring the limbal epithelium, the OCT can also provide an *in vivo* visualization of the palisades of Vogt. This is possible even in eyes where the palisades are not visualized clinically (41). Although the IVCM can also image the palisades, the image procurement takes time and requires a skilled and experienced operator whereas the process is much simpler in case of an OCT. Also, as seen with IVCM, in eyes with partial LSCD the thinning of the limbal epithelium is similar in the affected and unaffected areas (39). This epithelial thickness correlates with the presence of the palisades with significant thinning manifesting when the palisades are absent (42). Volumetric scans of the limbus provide a three dimensional image which can further help quantify the severity of LSCD (43, 44).

Scans from an AS-OCT can be subjected to image processing and thus the epithelial and stromal reflectivity is derived. Varma et al. found the epithelial reflectivity value to be a better indicator of the presence of LSCD than stromal reflectivity (45). They also studied the ratio of these two reflectivities (ES ratio) and proposed a cut off 1.29 to be diagnostic of LSCD with good sensitivity and specificity. Furthermore, a reversal of this ratio following SLET was noted by Kate et al. (12). However the values at the end of one year follow up did not reach the ES ratio seen in normal eyes (12).

#### OCT Angiography (OCT-A)

**Figure 4:**
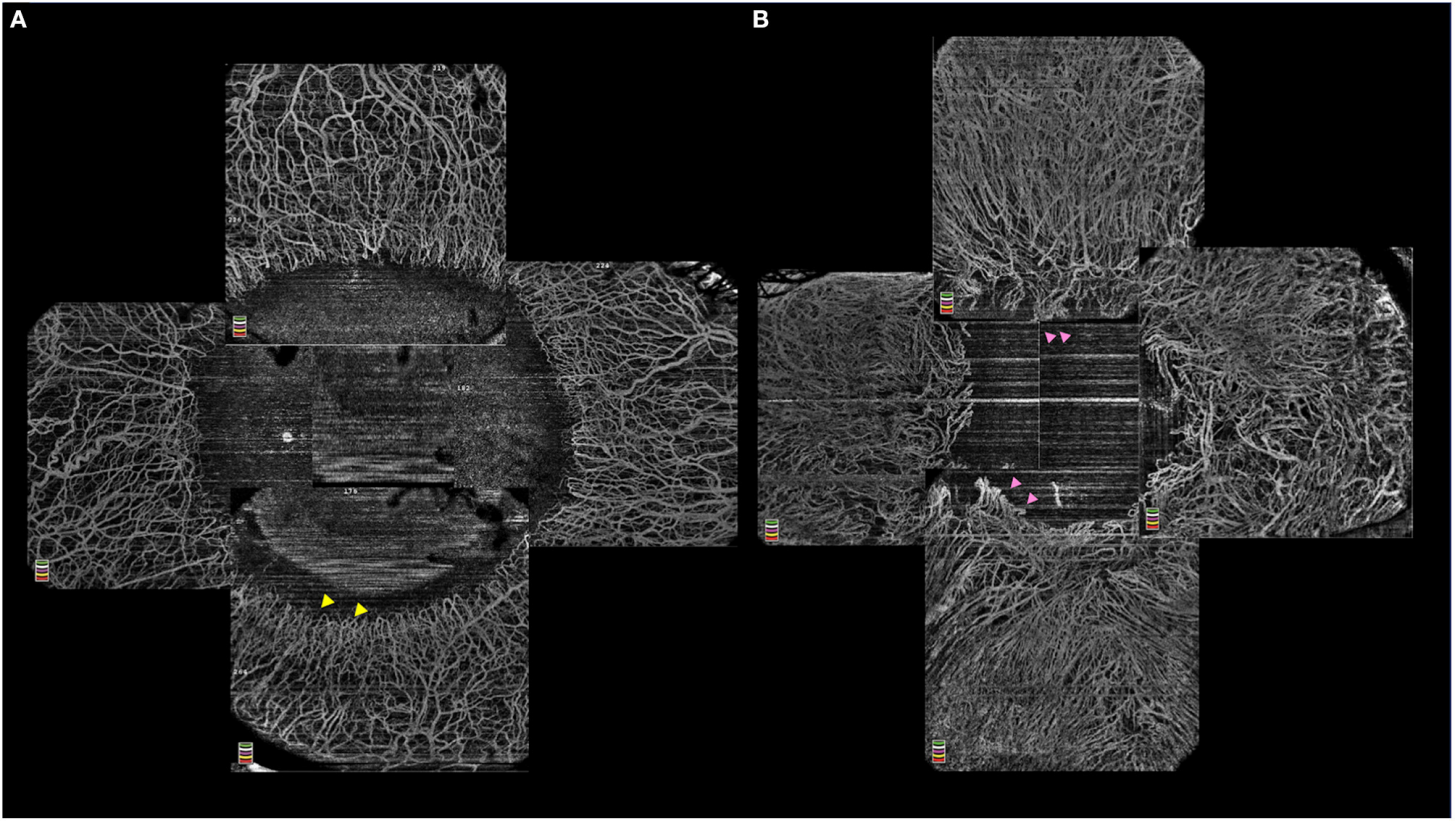
**(A)** Optical coherence tomography-angiography (OCT-A) illustrating a normal limbal vasculature with hairpin looped limbal vessels (yellow arrowheads) and surrounding normal perilimbal conjunctival and episcleral vessels. **(B)** OCT-A in in limbal stem cell deficiency with vascular invasion of the peripheral corneal and distortion of the annular ring of hairpin looped limbal vessels (pink arrowheads).

The use of the angiography feature of the OCT has been explored in quantifying the changes seen in the limbal vasculature as well as in corneal neovascularization (Figure 4) (46, 47). A progressive increase in the density of vascularization and its extent into the cornea has been reported with increasing severity of LSCD (48). Also, OCT-A has been used to differentiate true LSCD from its mimickers which also have corneal vascularization. A significant reduction in vascular density is noted after segmentation of the superficial layers in non-LSCD cases as in these eyes the vessels are usually located within the deep stromal layers (45). When this superficial vascular density values are >0.38, the diagnosis of LSCD can be confirmed with a sensitivity and specificity of 97.9% and 73.8% respectively (45).

## Classification

Several classifications have been proposed to grade the severity of LSCD (1, 2, 31, 49). These are based on corneal epithelial thinning, fluorescein staining patterns, presence of neovascularization, fibrovascular pannus, etc. The grading proposed by the Limbal Stem Cell Working Group has divided the corneal involvement into three groups depending on involvement of the central 5 mm of the cornea and these groups have further been subcategorized based on the percentage of limbal involvement (7). These gradations which are based on corneal findings help understand disease severity and assess progression. This is particularly helpful for uniform and standardized documentation for research and monitoring progression or response to therapy. However, the classification does not include adnexal involvement, and this is vital in the decision-making process for the management of these eyes. Hence, classification systems that incorporate the eyelid and conjunctival changes in addition to the corneal ones may better help in delivering appropriate therapy based on the composite disease severity (50).

## Management

The management of LSCD includes several surgical and non-surgical options and for each patient the treatment plan has to be tailored to suit the involved eye. However, LSCD rarely occurs in isolation and so the concurrent management of the systemic and ocular comorbidities is vital and often has to precede the surgical management of the disease. This includes systemic immunosuppression in cases of MMP, ocular anti-inflammatory therapy in cases of vernal keratoconjunctivitis, SJS, etc. A component of aqueous deficiency dry eye (ADDE) is usually present in most of these eyes and addressing the same with preservative free lubricants, punctal occlusion, etc. will aid in stabilizing the tear film prior to the surgical intervention.

**Figure 5:**
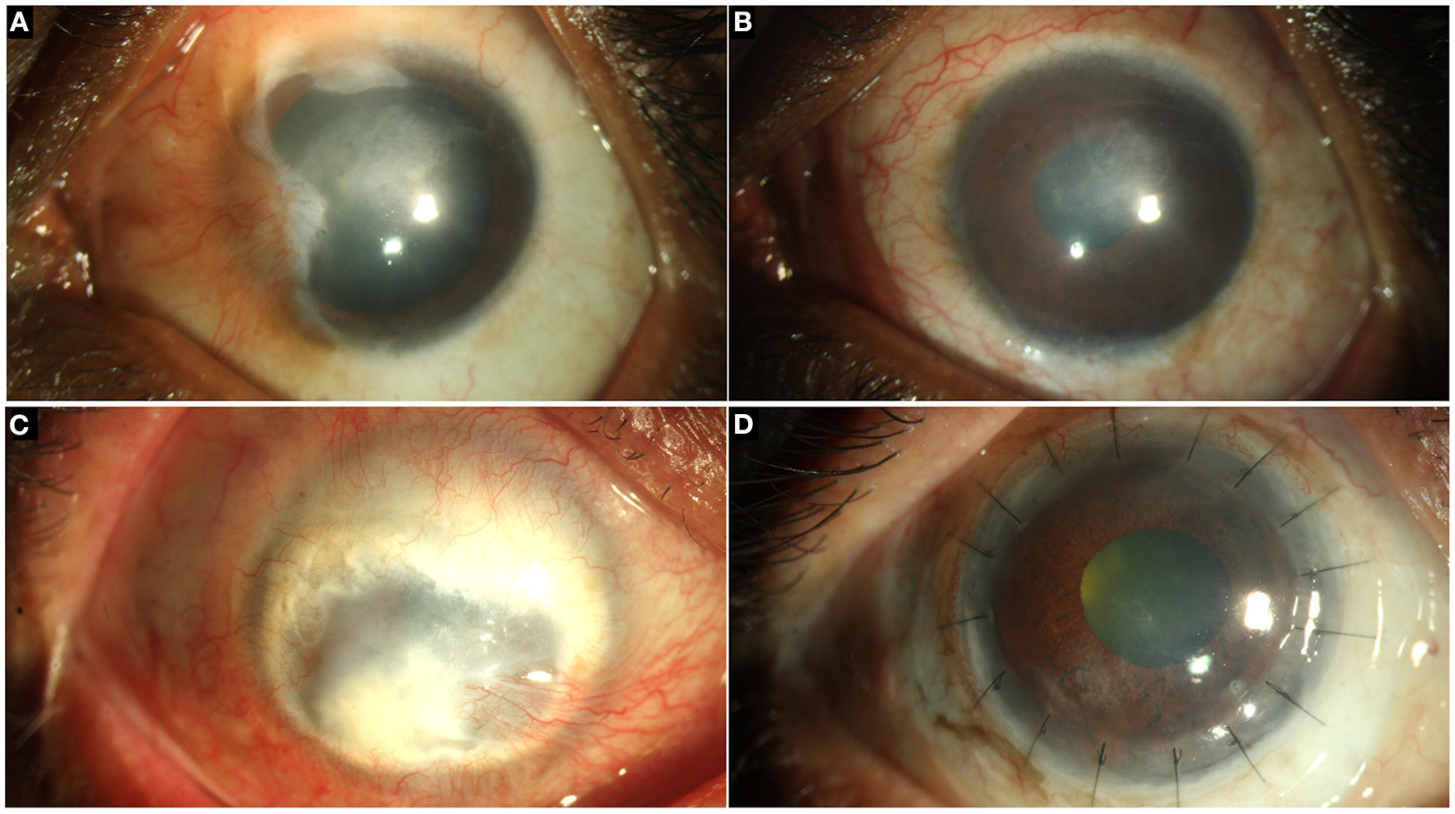
**(A)** Partial limbal stem cell deficiency (LSCD) following chemical injury managed with conjunctival limbal autograft (CLAu). **(B)** Restoration of a stable ocular surface is noted. **(C)** Total LSCD with leucomatous corneal scarring. **(D)** Reestablishment of an optically clear visual axis and a stable corneal epithelium with deep anterior lamellar keratoplasty and CLAu.

Several of the comorbidities present with LSCD also require surgical intervention and the sequence of these surgeries often determines the final functional outcome. Ideally, lid and other adnexal issues are addressed prior to the stem cell deficiency. In the presence of significant corneal scarring there is often need for a keratoplasty for visual rehabilitation (Figure 5). Although LSCT contributes to stromal remodeling and eventually a decrease in the density of the scar is noted, the degree to which this happens may vary. And so, several of these cases ultimately require a partial or full thickness corneal transplantation to restore an optically clear visual axis.

**Figure 6:**
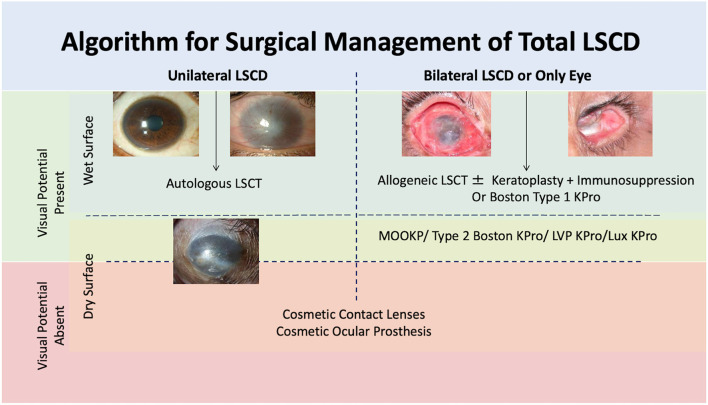
Algorithmic approach of management of limbal stem cell deficiency (LSCD). LSCT: limbal stem cell transplantation, KPro: keratoprosthesis, MOOKP: modified osteo-odontokeratoprosthesis.

The management of LSCD can be surgical or non-surgical depending upon the severity of damage to the LESC and the underlying pathology. Based on the clinical presentation, an algorithmic approach can be considered in most of the cases of LSCD (Figure 6).

### Partial LSCD

In cases of partial LSCD, the decision of surgical intervention is dictated by the involvement of the visual axis (Figures 2A-C). If the visual axis is affected, a surgical therapy is required is most cases. However, if the axis is clear, the patient can be followed up at regular intervals to determine if the disease is progressive or stationary. In case of the former, again the eye will require a surgical procedure while in case of the latter the same can be deferred.

#### Non-surgical Intervention

Eyes with partial LSCD with sparing of the visual axis and documented non-progression of the disease can be observed with regular follow ups. These cases can be visually rehabilitated with glasses or with rigid contact lenses when significant irregular astigmatism is present. Scleral lenses with large vaults are particularly beneficial in such eyes as they provide a fluid layer which addresses the dry eye component in addition to improving the visual acuity (51–53). Lenses which vault over the limbus are preferred as mechanical compression and trauma to the limbal epithelium is prevented (53). Optimizing the fit of the lenses in eyes with LSCD is vital as the resultant hypoxia in eyes with a compromised fit can exacerbate the severity of the LSCD (54).

#### Surgical Intervention

When partial LSCD is progressive or involving the visual axis, a surgical procedure is usually carried out to correct the same. The choice of procedure depends upon the involvement of the fellow eye. In unilateral cases an autologous LSCT is preferred where the LESC can be harvested from the contralateral eye or from the uninvolved areas of the same eye. In a comparative series with 70 patients, the outcome in eyes where the LESC were harvested from the same eye was similar to the outcome of eyes with stem cells from the contralateral eye (55). In bilateral cases also an autologous LSCT can be considered if the involved areas are limited to 3-4 clock hours in both eyes (56). Several studies have described the use of an amniotic membrane (AM) alone in the treatment of partial LSCD (57–62). Most of these reports have combined a superficial keratectomy to remove the conjunctival epithelium prior to placing the AM. Although the initial corneal epithelialization rates are good, the ability of the AM to maintain a stable epithelial surface in the long run is poor (58–61, 63). And so, an AM can be used for the temporary restoration of the ocular surface, until a LSCT can be performed. The use of only conjunctival autografts (CAG) has also been described in the treatment of partial LSCD. Shanbhag et al. found a better anatomical success rate with CAG when compared to LSCT in eyes with partial unilateral LSCD (64). Following the treatment of the LSCD, these patients may eventually require rigid contact lenses for visual rehabilitation.

### Total LSCD

In eyes with total LSCD, the initial step to determine the therapeutic approach would be to assess the presence of visual potential (Figure 6). In eyes with no visual potential, no further intervention is carried out unless there is a need to restore cosmesis in which case a contact lens trial is given, or an ocular prosthesis is implanted. In the presence of visual potential, the status of the fellow eye determines the next course of treatment.

#### Unilateral Total LSCD

In unilateral cases, if the surrounding adnexa is relatively uninvolved and the ocular surface is wet with a fairly clear corneal stroma, an autologous LSCT is performed. If there are significant cicatricial changes of the conjunctiva, a combined or staged procedure with a conjunctival autograft (in unilateral cases) or mucous membrane graft (in bilateral cases) can be planned (65). Similarly if a lamellar or penetrating keratoplasty (LK or PK) is planned for visual rehabilitation, it can carried out as a one or two step procedure (66–69). Although the grafts maintain clarity in the initial postoperative period after a combined procedure, the rate of rejection is usually higher in these cases and so a staged procedure is preferred (67–70). Whenever possible a LK is favored over a PK as the former lacks a transplanted endothelium and so is associated with lower rates of rejection.

#### Bilateral Total LSCD

The treatment algorithm for bilateral cases is similar to that of unilateral cases (71). If no dry eye is detected and the conjunctiva and lids are relatively uninvolved, then an allogeneic LSCT is the chosen procedure. In the presence of significant symblephara with adnexal pathologies the choice of LSCT over keratoprosthesis (KPro) depends upon the surgeon's preference. The former will require multiple procedures to correct the co-morbid pathologies before the LSCD is addressed. Systemic immunosuppression will also be necessary in view of the allogeneic nature of the transplant. A keratoprosthesis will circumvent these issues and offers a one-step procedure with early visual rehabilitation (72). Nevertheless, this technique is associated with several serious sight threatening complications such as glaucoma, retinal detachment, implant extrusion, endophthalmitis, etc (73–75). Thus, KPros are usually reserved for eyes with end stage corneal pathologies or in eyes where prior LSCTs have failed (76).

**Figure 7:**
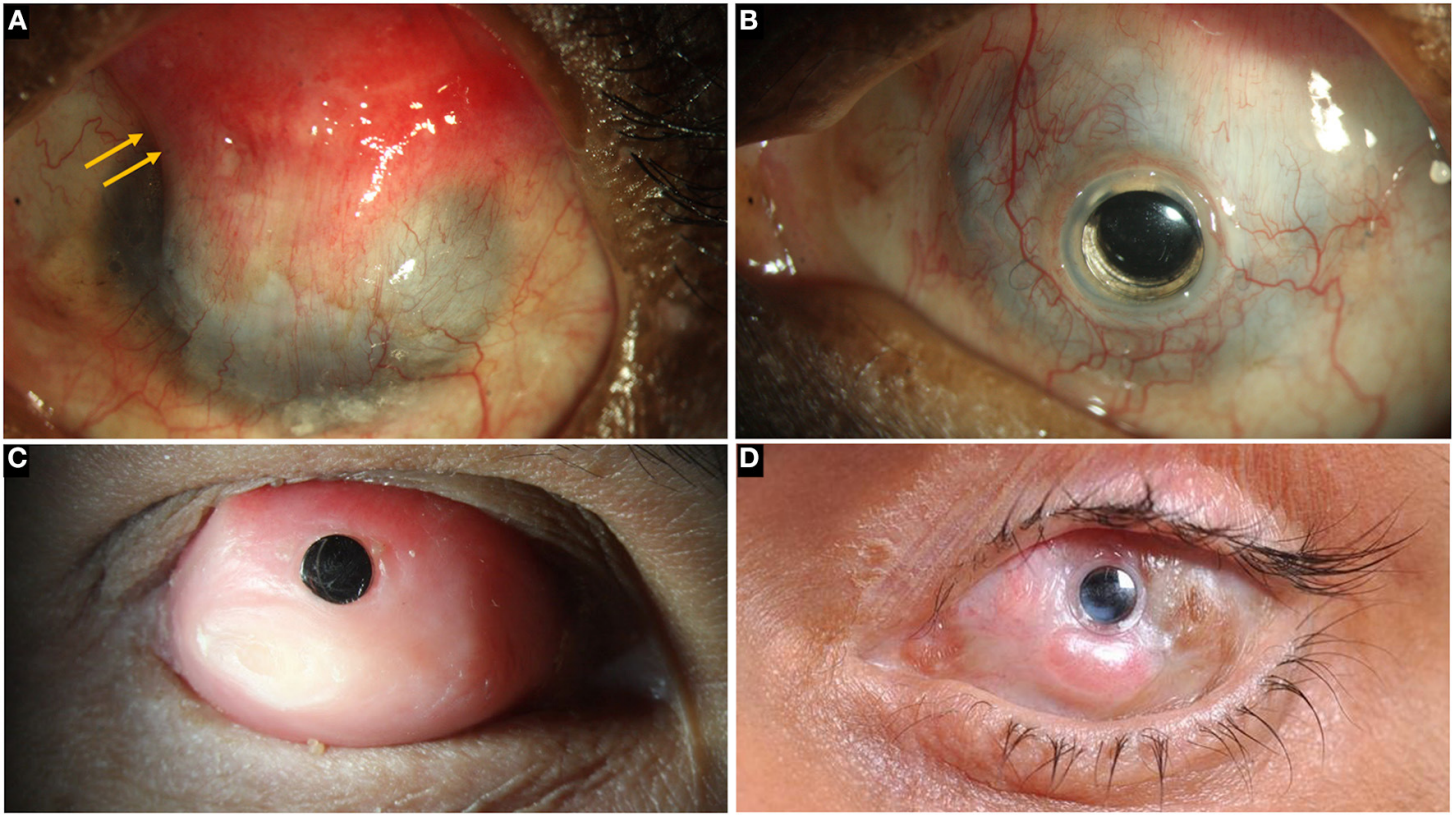
**(A)** Left eye in a case of bilateral total limbal stem cell deficiency (LSCD) with a wet surface due to Stevens-Johnson syndrome (SJS). A superior conjunctival hooding (yellow arrows) was carried out previously for microbial keratitis with a corneal perforation. **(B)** A Boston keratoprosthesis in the same eye. **(C)** Modified osteo-odontokeratoprosthesis in an eye with total LSCD and a dry ocular surface. **(D)** LVP KPro in an eye with SJS.

There are different types of KPros and the choice of one KPro over the other is determined by the presence or absence of ADDE. Table 2 lists different types of KPros that have been utilized in the management of LSCD. If the surface is wet, a Boston KPro type 1 or Aurolab KPro (auroKPro) is carried out and if the eye has ADDE, then a Boston KPro type 2, LV Prasad KPro (LVP KPro) or modified osteo-odontokeratoprosthesis (MOOKP) is performed (Figure 7). The Boston KPro type 1 is the most commonly used prosthesis and has an optical cylinder with a skirt of donor cornea (Figures 7A,B). It has good visual outcomes and retention rates especially in eyes with non-autoimmune underlying diseases (74, 75, 77, 92–94) Since the cost of the device is a major inhibitory factor for its use, the auroKPro, its cheaper alternative is a more viable option in low resource settings. Both prosthesis have similar outcomes in terms of visual function, retention rates, and other secondary complications (95, 96).

**Table 2:** Brief description of various KPros employed in the management of limbal stem cell deficiency.

	**Type of Keratoprosthesis**	**Structure**
Biocompatible KPro	Boston KPro 1 (77)	PMMA optical cylinder fitted with a titanium back plate. Complex is secured with a titanium locking ring
	Boston KPro 2 (78)	Similar to Boston KPro 1-has an additional anterior PPMA segment which projects through the lids
	Auro KPro (79)	Similar to Boston KPro 1 but with a PMMA backplate
	LUX (80)	PMMA optic, titanium backplate and a titanium sleeve
	LVP KPro (81)	Similar to Boston KPro 1 but with a longer optical cylinder which allows tucking of MMG beneath the front plate
	S-KPro (82, 83)	PMMA optic with a polyurethane and polypropylene skirt.
	Lucia KPro (84)	Boston KPro with reduced manufacturing cost by altering the design of the backplate
	Filatov KPro (85)	Titanium frame with two flanges with a PMMA cylinder
	Fyodorov–Zuev KPro (86)	Similar to MICOF KPro but implanted in a single sitting
	MICOF KPro (87)	Titanium frame with two flanges within which a PMMA cylinder is threaded. Auricular cartilage is also used to supplement the implant
Bio-integrable KPro	Pintucci KPro (88)	Central PMMA optic with a peripheral Dacron skirt
	AlphaCor (Chirila KPro) (89)	Made of poly-2-hydroxyethyl methacrylate with different water content in the central clear optical zone and peripheral bio-integrable skirt
	Legeais BioKPro-III	Polytetrafluoroethylene skirt and polyvinylpyrrolidone-coated polydimethylsiloxane optic
Biological KPro	MOOKP (90)	Optical cylinder is embedded in the canine tooth and implanted in a bed of MMG over the ocular surface
	Osteo-KPro (91)	Similar to MOOKP-tibia is used instead of a tooth

*KPro, keratoprosthesis; MOOKP, Modified osteo-odonto keratoprosthesis; S-KPro, Seoul keratoprosthesis; MICOF, Moscow Eye Microsurgery Complex in Russia; LVP KPro, LV Prasad Keratoprosthesis*.

In case of dry eyes or dermalised ocular surfaces with lid changes, both Boston KPro type 2 and the MOOKP have good functional and anatomical outcomes (90, 97–99). The former is similar to its type 1 counterpart and has a longer cylinder which is exteriorized through lid while the latter has a cylinder embedded in an osteo-dental lamina (Figure 7C). However, the surgical procedure for both devices is cumbersome, time consuming and has a steep learning curve. The LVP KPro, which is similar to the Boston KPro with a longer optical cylinder, is implanted as a two staged procedure under a mucous membrane graft used to reconstruct the ocular surface (Figure 7D) (78, 100). Its anatomical outcomes are better than those of Boston KPro type 2 but they are not superior than those of MOOKP (78). Table 3 compares the outcomes of the most commonly used KPros in LSCD.

**Table 3:** Comparison of the most commonly used keratoprosthesis in the management of limbal stem cell deficiency.

**KPro**	Prerequisite*	**Number of surgeries required**	Outcomes**		
			***Follow up years***	***Retention rate %***	***Visual Recovery %***
Boston KPro Type 1 (95)	Wet ocular surface	1	5	74	51
AuroKPro (96)	Wet ocular surface	1	5	43	35
Boston KPro Type 2 (78)	Intact lids	1	5.9	50	38
LVP KPro (100)	-	2	2.5	76	36
MOOKP (99, 101)	Adults, healthy oral cavity	2	1	96–100	45–83^#^

* *Prerequisites in addition to being suitable for a KPro*.

** *Visual recovery is proportion of eyes with vision better than 20/200*.

# *Proportion of eyes with vision better than 20/60*.

*KPro, keratoprosthesis*.

Transplantation of cultivated oral mucosal epithelium (COMET) is another alternative in eyes with bilateral LSCD where labial or buccal epithelial cells are cultured on an AM and transplanted over the cornea. Studies have reported a stable ocular surface following the procedure however there is a higher risk of persistent epithelial defects, corneal neovascularization and graft rejection when compared to LSCT (81, 102–104). And so, an allogeneic LSCT is considered superior to and is favored over COMET despite the latter being an autologous transplant with no requirement for systemic immunosuppression (104). In a series comparing the outcomes of cell based therapies (CLET, CLAL, COMET) vs. Boston KPro type 1 in cases of bilateral LSCD without ADDE, the KPro group was found to have the best functional outcome at the end of five years (68, 71). However, a recent meta-analysis revealed that in patients undergoing LSCT, nearly 61% maintained a vision of at least 20/200 at end of 2.5 years which is similar to the 64% of patients who had the same vision in the KPro group (105).

Various modifications of the COMET procedure have been proposed which alter the type of carriers used to transfer the cultivated cells. These include the AM, fibrin glue and temperature sensitive polymers. In case of the latter, the polymer is stable at 37°C, however when the temperature drops to 30°C, the cultivated epithelial sheet detaches spontaneously (106, 107). This is in contrast to traditional methods where a carrier or enzymatic detachment is required. Furthermore, biomaterial free sheets have also been used, wherein the cultivated sheet is directly transplanted from the culture plate onto the eye without a carrier for the cells (108, 109). Establishment of a well epithelialized surface have been reported with the use of the same and these outcomes were found to be better than those of COMET with the use of AM as a substrate (108, 109).

As an alternative to cultivation of oral mucosal epithelial cells, which requires the necessary infrastructure, direct transplantation of the oral mucosa has also been described for the management of LSCD (110, 111). The graft is transplanted directly over the limbal area and can re-establish a stable surface and cause regression of neovascularization (110, 111). An additional benefit that the mucosal graft has over conventional LSCT is that adnexal pathologies such as lid margin keratinization or symblephara can be addressed with the same harvested tissue. As the procedure is autologous, no systemic immunosuppression is required. A similar approach has also been reported with the use of nasal mucosal grafts which primarily aim to replenish the goblet cells in the ocular surface (112).

### Technique of LSCT

#### Types

There are two chief types of LSCT: allogeneic and autologous. These can be further divided into different types based on the anatomical source of the graft which includes conjunctival limbal auto or allograft (CLAu and CLAL), allogeneic keratolimbal allograft (KLAL) or pure limbal tissues as in cases of auto and allogeneic cultivated or simple limbal epithelial transplants (CLET and SLET). In cases of allogeneic LSCT, the donor can be a cadaveric or a living related donor. In pure limbal transplants, once the limbal lenticule is harvested it can be directly transplanted as in SLET where the proliferation of epithelial cells occurs *in vivo* over the corneal surface. Alternatively, the biopsied tissue can be cultivated *in vitro* and then transplanted as a sheet of epithelium as in case of CLET.

#### Choice of Procedure

**Figure 8:**
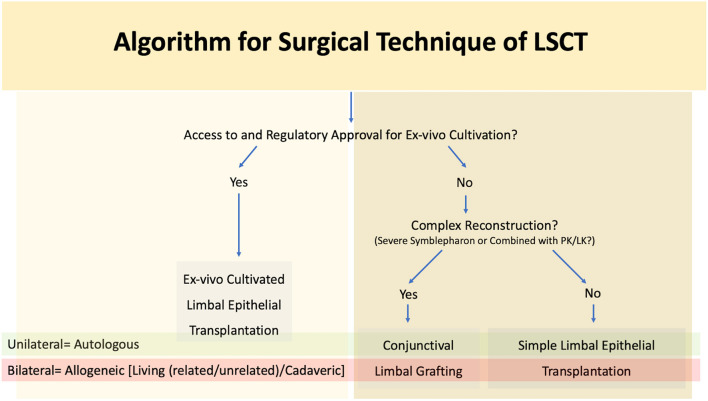
Algorithm for surgical technique of limbal stem cell transplantation (LSCT) PK, penetrating keratoplasty; LK, lamellar keratoplasty.

As mentioned previously autologous procedures are performed in unilateral cases while allogeneic transplants are reserved for bilateral LSCD (Figure 8). The major difference between the two lies in the need for long term systemic immunosuppression for allogeneic LSCT. A combination of corticosteroids and steroid sparing agents are usually given initially, and the patients are then maintained only on the steroid sparing immunosuppressive agent (113, 114) Most of these medications are both expensive and associated with a side effect profile necessitating regular systemic monitoring (113, 114).

**Figure 9:**
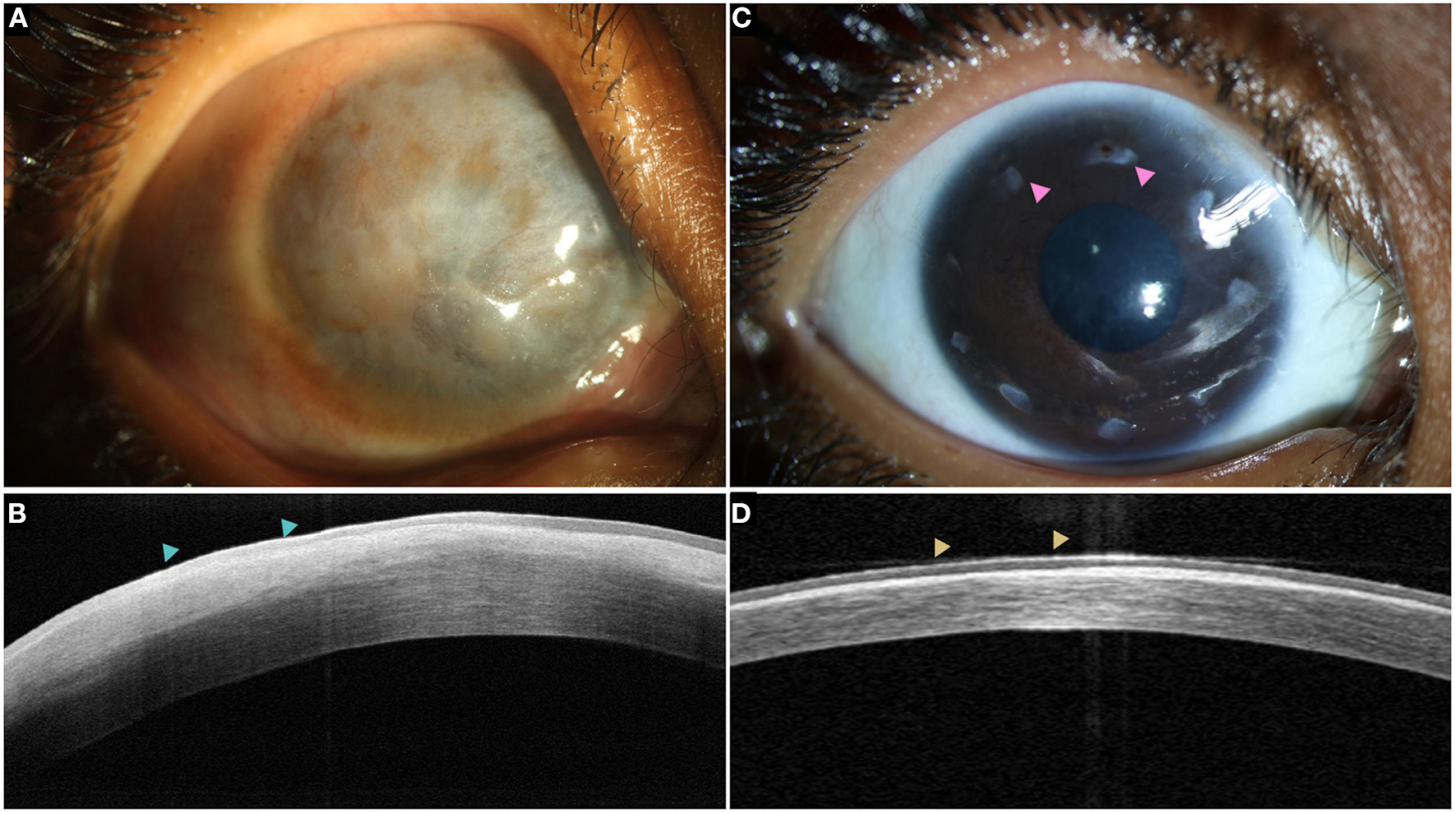
**(A)** Total LSCD with a thick pannus in a case of chronic vernal keratoconjunctivitis with hyperreflective epithelium (blue arrowheads) on the optical coherence tomography (OCT) line scan **(B)**. **(C)** A stable ocular surface is observed following allogeneic simple epithelial limbal transplantation. The intact limbal tissues are also visible (pink arrowheads). **(D)** Restoration of epithelium with a normal reflectivity is noted on the OCT scan (yellow arrowheads).

The choice of procedure is often determined by the extent of involvement of the surrounding adnexa. A limbal transplant (SLET/CLET for autologous cases, SLET/CLET/KLAL for allogeneic cases) is preferred for LSCD in wet eyes without significant adnexal involvement (Figure 9). Access to a laboratory facility with regulatory approval is required for the practice of cultivated stem cells. CLAu or CLAL is preferred in cases where concurrent correction of cicatricial conjunctival changes is also required as seen in eyes with significant symblephara adjacent to a partial LSCD (Figure 5). The graft can be harvested from the same eye or fellow eye, depending upon the amount of healthy residual limbus. In the traditional CLAu, a large limbal graft is usually harvested (4-6 clock hours) which can result in an iatrogenic LSCD. To avoid this complication, a mini-CLAu with only 1-2 clock hours of limbal tissue is a viable substitute (66, 115). Alternatively conjunctival tissue can be harvested separately as a CAG along with a pure limbal transplant (CLET/SLET). This combination is usually adopted in eyes with total LSCD and symblephara. Tables 4, 5 detail the relative advantages and disadvantages of each of the LSCT procedures.

**Table 4:** Comparison of different autologous Limbal stem cell transplantation procedures.

**Procedure**	**Regulatory approval**	**Laboratory set up**	**Risk of iatrogenic LSCD in donor eye**	**Feasibility of a repeat procedure**	**Number of procedures required**
SLET	Not required	Not required	No	Yes	1
CLET	Required	Required	No	Yes	2
CLAu	Not required	Not required	Yes	No	1
Mini-CLAu	Not required	Not required	No	Yes	1

**Table 5:** Comparison of different allogeneic Limbal stem cell transplantation procedures.

**Procedure**	**Regulatory approval**	**Laboratory set up**	**Need for immunosuppression**	**Feasibility of a repeat procedure**	**Number of procedures required**
SLET	Not required	Not required	Yes	Yes	1
CLET	Required	Required	Yes	Yes	2
CLAL	Not required	Not required	Yes	No	2
KLAL	Not required	Not required	Yes	Yes	1

*SLET, simple limbal epithelial transplant; CLET, cultivated limbal epithelial transplant; CLAu, conjunctival limbal autograft; CLAL, conjunctival limbal allograft; KLAL, keratolimbal allograft*.

#### Comparison of Outcomes

In a systematic review of 1023 eyes, SLET and CLAu were found to have better outcomes than CLET in cases of unilateral LSCD (116). A similar result was seen in a recent meta-analysis where SLET was found to have better functional outcomes when compared to CLET (117). The overall performance of autologous procedures has been deemed to be better than that of allogeneic procedures with the latter having a failure rate of up to 40% (105). The former group of procedures also have a higher percentage of patients with a 2 line improvement in visual acuity following surgery (105).

Ganger et al. found CLET and KLAL to have similar anatomical outcomes, but KLAL fared better than CLET in terms of functional outcomes (117). The cumulative success of KLAL from a systematic review was found to be 63% with 69% of cases having vision better than 20/200 (118). A recent series on allogeneic SLET reported a success rate of 83% and more than 60% of the cases had an improvement in vision which was >20/60 (119). And so, in the context of the expensive nature of CLET with its need for a laboratory set up, KLAL and allogeneic SLET are perhaps the more feasible options in cases of bilateral LSCD. However more studies are required on the long-term outcomes of allogeneic SLET to determine its benefits over other allogeneic procedures. Table 6 compares the outcomes of different modalities of stem cell transplants.

**Table 6:** Comparison of indications and outcomes of different surgical modalities of management of limbal stem cell deficiency.

**Surgical Procedure**	**Tissue transplanted**	**Indication**	Outcomes*	
			***Anatomical %***	***Functional %***
SLET (120)	LESC	Autologous, Allogeneic LSCD	78, 83	69, 60
CLAu (117)	Conjunctiva+LESC	Autologous LSCD	81	74.4
CLAL/KLAL (118)	Conjunctiva+LESC	Allogeneic LSCD	68	51
CLET (106)	Limbus	Autologous, Allogeneic LSCD	71, 52	65, 65
COMET (102)	Oral mucosal epithelium	Allogeneic LSCD	71	64
Oral mucosa transplantation (112)	Oral mucosa	Allogeneic LSCD	86	71
Nasal mucosa transplantation (113)	Nasal mucosa	Allogeneic LSCD	NA	18

*SLET, simple limbal epithelial transplantation; CLET, Cultivated epithelial limbal transplantation; COMET, Cultivated oral mucosal epithelial transplantation; LSCD, limbal stem cell deficiency*.

* *Anatomical outcomes: defined as a stable, avascular surface*.

*Functional outcome: proportion of eyes with vision better than 20/200*.

### Recent Advances

The search for new therapies for LSCD is always ongoing because of the need for treatment modalities that do not have the risk of rejection, require immunosuppression, etc. And the epitome of such endeavors would be to arrive at a medical therapy for LSCD. One such intervention was identified serendipitously during the treatment of patients with ocular surface neoplasia with interferon α-2b and retinoic acid (120). These cases had partial LSCD which responded to the topical medications. The rationale proposed for the same was that retinoic acid improves corneal wound healing and promotes proliferation of transient amplifying cells while interferon α-2b mediates the healing through its anti-inflammatory function, specifically on macrophages (120).

Another novel technique in the treatment of total LSCD is the amnion-assisted conjunctival epithelial redirection (ACER) which involves the placement of an amniotic membrane over the cornea and limbal explants. The edges of the membrane are tucked under the free edges of the recessed conjunctiva and as a result of this, the conjunctival cells migrate over the membrane (121). This allows the limbal explants under the membrane to proliferate over the surface of the cornea unhindered. Establishment of a stable ocular surface has been reported following this procedure. The use of a modified version of this procedure has also been described for partial LSCD with good outcomes (122).

Novel prosthetic devices such as the Lux and CorNeat keratoprosthesis are being developed as alternatives to LSCT. The former is similar to a traditional Boston KPro with a polymethylmethacrylate cylinder and a titanium backplate (123). This prothesis does not rely on the presence of intact lids which is required for Boston KPro type 2 and has better cosmesis than a MOOKP. Thus the Lux KPro is a viable option for eyes with dry ocular surfaces and LSCD, with good functional vision and retention rates (123). The long term outcomes with this device are awaited. The CorNeat is a true corneal prosthetic device and is structurally different from other KPros. This synthetic cornea has a central PMMA optic and a surrounding porous skirt made of polyurethane fibers (80). The skirt is implanted beneath the conjunctiva where it integrates with the surrounding tissue. Animal models with the CorNeat KPro have shown good retention of the implant while results of human trials are awaited (80).

The use of stems cells obtained from sources other than the LESC is another interesting avenue being explored in the management of LSCD. Of these, limbal mesenchymal stem cells have been best studied and have an established role in corneal wound healing, scar remodeling and angiogenesis (124–127). Its role as a therapeutic option for LSCD is being investigated with a recent clinical trial suggesting that they are as efficacious as CLET in restoring a stable ocular surface (128). Other stem cells that are being studied include those from hair follicles, dental pulp, embryonic stem cells, etc (129–133). Their exact utility and efficacy in LSCD is yet to be determined.

## Summary

This review presents an overview of the different diagnostic tests and management modalities in LSCD in order to provide a clinical perspective which will help the physician determine the best course of therapy in cases with LSCD. An in-depth write-up on the pathophysiology of stem cell deficiency is beyond the scope of this review. The diagnosis of limbal stem cell deficiency is often made based on clinical features but can be supplemented by several investigative tools especially when faced with challenging case scenarios. Although both impression cytology and IVCM can confirm the diagnosis of LSCD the expense of the equipment involved, and the skilled personnel required often restrict their use. AS-OCT is a more commonly available device and has several measurable parameters which can be used in the diagnosis of LSCD. However more studies are required to determine the exact diagnostic cut offs. The interpretation of the results of any of these tests must be made in the context of the clinical picture to arrive at the correct diagnosis. Additionally, these investigative modalities have also been used to monitor the response to LSCT and to confirm the restoration of a corneal epithelial phenotype (10, 134–136). Using a combination of clinical and one or more diagnostic tests, a standardized method of validating the outcomes of LSCT can be established.

A comprehensive approach is usually required for the management of LSCD with simultaneous treatment of comorbid ocular and systemic pathologies. Autologous LSCT for unilateral LSCD and allogeneic LSCT for bilateral cases, in the absence of dry eye, are the preferred modalities of therapy which render a stable ocular surface and good visual outcomes. A KPro is favored in more complex cases and provides a rapid visual recovery. The exact choice of procedure is ultimately dependent upon the status of the adnexa, the resources available and the expertise of the surgeon.

## Author Contributions

AK contributed to the collection of resources, original draft preparation, and revisions of the manuscript. SB contributed to the conceptualization, methodology, supervision, revision, and editing of the manuscript. Both authors contributed to the article and approved the submitted version.

## Funding

Hyderabad Eye Research Foundation.

## Conflict of Interest

The authors declare that the research was conducted in the absence of any commercial or financial relationships that could be construed as a potential conflict of interest.

## Publisher's Note

All claims expressed in this article are solely those of the authors and do not necessarily represent those of their affiliated organizations, or those of the publisher, the editors and the reviewers. Any product that may be evaluated in this article, or claim that may be made by its manufacturer, is not guaranteed or endorsed by the publisher.
